# Textural Features of MR Images Correlate with an Increased Risk of Clinically Significant Cancer in Patients with High PSA Levels

**DOI:** 10.3390/jcm12082836

**Published:** 2023-04-12

**Authors:** Sebastian Gibala, Rafal Obuchowicz, Julia Lasek, Zofia Schneider, Adam Piorkowski, Elżbieta Pociask, Karolina Nurzynska

**Affiliations:** 1 Urology Department, Ultragen Medical Center, 31-572 Krakow, Poland; 2 Department of Diagnostic Imaging, Jagiellonian University Medical College, 31-501 Krakow, Poland; 3 Faculty of Geology, Geophysics and Environmental Protection, AGH University of Science and Technology, 30-059 Krakow, Poland; 4 Department of Biocybernetics and Biomedical Engineering, AGH University of Science and Technology, 30-059 Krakow, Poland; 5 Department of Algorithmics and Software, Silesian University of Technology, 44-100 Gliwice, Poland

**Keywords:** prostate cancer, MRI, PSA, textural analysis, multiple-instance learning, support vector machine

## Abstract

Background: Prostate cancer, which is associated with gland biology and also with environmental risks, is a serious clinical problem in the male population worldwide. Important progress has been made in the diagnostic and clinical setups designed for the detection of prostate cancer, with a multiparametric magnetic resonance diagnostic process based on the PIRADS protocol playing a key role. This method relies on image evaluation by an imaging specialist. The medical community has expressed its desire for image analysis techniques that can detect important image features that may indicate cancer risk. Methods: Anonymized scans of 41 patients with laboratory diagnosed PSA levels who were routinely scanned for prostate cancer were used. The peripheral and central zones of the prostate were depicted manually with demarcation of suspected tumor foci under medical supervision. More than 7000 textural features in the marked regions were calculated using MaZda software. Then, these 7000 features were used to perform region parameterization. Statistical analyses were performed to find correlations with PSA-level-based diagnosis that might be used to distinguish suspected (different) lesions. Further multiparametrical analysis using MIL-SVM machine learning was used to obtain greater accuracy. Results: Multiparametric classification using MIL-SVM allowed us to reach 92% accuracy. Conclusions: There is an important correlation between the textural parameters of MRI prostate images made using the PIRADS MR protocol with PSA levels > 4 mg/mL. The correlations found express dependence between image features with high cancer markers and hence the cancer risk.

## 1. Introduction

Prostate cancer is the second most frequently diagnosed cancer in men, with approximately 1.4 million diagnoses worldwide in 2020 [1,2]. The basic methods used in the early diagnosis of prostate cancer are laboratory tests, and, in particular, the determination of the PSA tumor marker. Since its discovery in 1979 and through its clinical use in the 1980s and 1990s, the prostate-specific antigen (PSA) has evolved into an invaluable tool for the discovery, assessment, and monitoring of prostate cancer in men. Increased serum PSA levels are likely a product of disturbed cellular architecture in the prostate gland [3]. PSA is believed to correlate highly with prostate disease; it is produced by many glands but is also associated with non-malignant diseases [4]. Moreover, the influence of hormonal therapy influences the detection of prostate cancer [5]. After the introduction of MR scanners to medical practice, the first attempt to diagnose prostate glands was made by Damadian in the early 1970s; ten years later, it was recognized as a valuable tool for the process of diagnosing prostate diseases [6,7]. However, the cost of these examinations has raised some doubts [8] as to overdiagnosis [9]; the role of MR is well established in the process of diagnosing prostate diseases [10], especially after the introduction of diffusion-weighted techniques and contrast enhancement to the protocol [11,12]. The experience gained over the years of using of MRI techniques in prostate imaging, especially where correlated with biopsies [13], resulted in the formation of imaging standards, PIRADS 1 and PIRADS 2, where the PIRADS 4 and PIRADS 5 stages are linked to a high probability of the presence of cancer and associated with high PSA levels [14]. MRI has taken on an increasingly significant role in the diagnostic process with the development of computed systems for image segmentation and analysis, and algorithms for prostate tumor detection have been proposed [15].

However, manual analysis is laborious and dependent on well-trained specialists in the field of diagnostic imaging. Therefore, efforts have been made to develop a computer-aided diagnosis (CAD) tool. It has been proven that automatic approaches improve the specificity, as compared to moderately experienced radiologists, for more difficult tumors [16] and allow for global standardization across radiological centers [17]. The described CAD systems use various modalities, from T2-weighted MRI (T2W-MRI) [18] to prostate biomarkers, and diffusion-weighted MRI (dw-MRI) supported with level-sets methods for prostate delineation [19].

Grayscale MR images are the sum of pixels of different brightness and grayscale levels. Patterns formed by sub-sets of similar pixels can be differentiated with the use of texture maps where pixel distribution changes can be characterized with the application of mathematical formulas [20,21,22]. A plethora of textural features that cannot be recognized by the human eye can be characterized on the basis of mathematical analyses. This encoded information can be revealed by texture feature maps [22].

Magnetic resonance imaging (MRI) is a non-invasive method of inspecting internal bodily structures. The high quality of the gathered data allows specialists to determine whether the observed tissue corresponds to clinically significant or non-significant prostate cancer [23,24].

Over time, the use of artificial intelligence (AI) methods to enable the creation of a CAD system has been widely investigated. In their review, Booven et al. [25] focus on artificial neural networks (ANNs) as a method of analyzing a variety of data connected with prostate cancer detection.

Starting with prostate-specific antigen (PSA) value analysis, rather than other medical markers, and developing an understanding of MRI images, biomarker diagnosis, histopathological data utilization, etc., the authors conclude that deriving textural features is the most promising approach for prostate cancer description.

Several other reviews support this claim. For instance, Harmon et al. [26] show that AI enables a correlation between pathological and radiological information about the patient when multiparameter MRI (mp-MRI) is considered. Describing the mp-MRI with radiomics (quantitative imaging of features that are poorly recognized by the human eye, but are understandable for computers) supported with AI algorithms allows for the prediction of cancer aggressiveness [27] or general prostate cancer analysis [28]. The influence of deep learning models, which have recently gained popularity, on healthcare professionals working with MRI has been investigated by Liu et al. [29]; meanwhile, Wildeboer et al. [30] concentrated on building a CAD system with this technology.

A significant amount of research addresses the problem of determining the Gleason score using the textural features calculated for various modalities of MRI. Wibmer et al. [31] build a binary classifier using Haralick textural features [32] for the determination of cancerous and non-cancerous prostate images. A similar approach shows that textural features derived from T2W-MRI, dw-MRI, and dynamic contrast-enhanced MRI (DCE) effectively distinguish the Gleason scores [33,34,35]. More recent research that is still based on first- and second-order histogram features distinguishes up to five grades in the Gleason Grade Group [36,37].

Radiomics features are used to determine the aggressiveness of prostate tumors [38,39]. When calculated for types of mp-MRI including T2W-MRI, dw-MRI, apparent diffusion coefficient imaging (ADC), and DCE, its performance is comparable with the Prostate-Imaging Reporting and Data System (PIRADS) [40,41,42]. On the other hand, when radiomics features are supported by additional medical risk factors, a model based on multivariate logistic regression allows for the classification of clinically significant and non-significant prostate cancers [43,44]. Using mp-MRI data for feature calculation is justified in previous research where support vector machine (SVM) models based on mp-MRI outperformed those constructed using T2W-MRI or ADC data only.

Recently, deep learning approaches have become a popular method of designing models for building a CAD system [45], detecting and grading prostate cancer [46], and differentiating clinically significant and non-significant prostate cancers [47,48]. In the last case, the performance was comparable to that of the PIRADS system.

There are also methods for automated cancer detection based on T2W-MRI data only [49,50], which perform the automatic grading of prostate biopsy specimens in correlation with Gleason grades based on SVM models [51]. There are also solutions based on textural features derived from mp-MRI for detecting transition zones in prostate tumors [52], and models that use bi-parametric MRI texture analysis for detecting and evaluating high-grade prostate cancer [53].

## 2. Materials and Methods

### 2.1. Method Overview

The study protocol was developed according to the Declaration of Helsinki and the Declaration of Good Clinical Practice [54]. All images were anonymized prior to processing to ensure the security of personal data. In addition, written consent from the Local Ethics Committee was obtained to conduct this study, 1072.6120.21.22 (23 February 2022). The study used data from 125 patients aged 27 to 87 years. MRI images were acquired (1.5 T Siemens Avanto, Enlargen, Germany) during normal diagnostic procedures, which met the standard of the PIRADS protocol with the presence of T2-weighted axial sequences with 2 mm slices and a distance factor of 0 and diffusion-weighted sequences with the use of a single shot echo planar sequence (EPI) made with b value equal to 0–800–1500 and a distance factor of 0. Finally, T1-weighted sequences were designed for post-contrast evaluation. The parameters of the T2-weighted sequence utilized in the present study are summarized in Table 1. Images were converted from 12 bits to 8 bits by linearly scaling from min to a range of 0–255. Then, images that met the stable (repeatable) conditions were selected for the analysis of the texture features. After analyzing clinical data, information on PSA value was obtained for 41 patients (values 0.39–19 ng/mL) (see Appendix A).

In the present work, we aimed to verify whether it is possible to distinguish with a high probability between patients with prostate cancer and healthy patients. The content of T2W-MRI scans was analyzed, while the PSA was the reference. For the MRI scans, the textural features were calculated using the MaZda software. For the data processing, manually annotated masks were used. As a result, the texture features were calculated for three different regions: the inner prostate only, the outer prostate only, and the inner and outer prostate regions treated as a whole. In order to create a binary classification model, multiple-instance learning (MIL) with a support vector machine (SVM) was adopted. This approach corresponds well with the characteristics of the collected dataset, where one PSA value is known for a patient described with several MRI scans. At the same time, the cancerous tissue may be visible only in one part. It was assumed that PSA = 1, 2, 3 correspond to a healthy person (label = 0) and PSA ≥ 4 is for an ill one (label 1). Finally, the leave-one-out methodology was applied.

### 2.2. Prostate MRI Dataset

**Figure 1:**
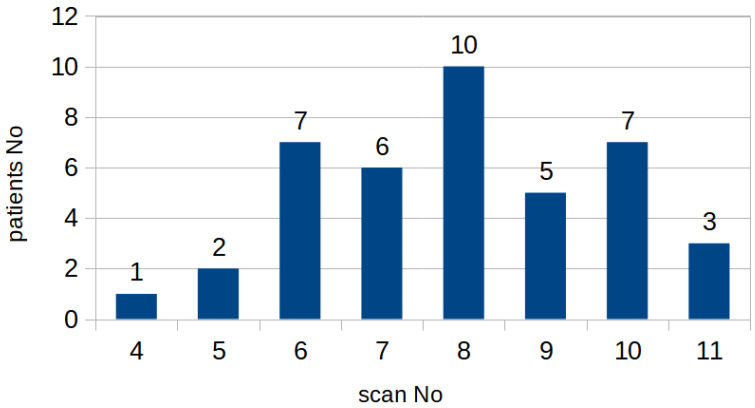
Histogram of the number of patients with similar numbers of MRI scans. The number of analyzed images—slices from different patients varied due to gland size and zone margins. Figure presents groups of patients with different number of slices considered in the study.

**Figure 2:**
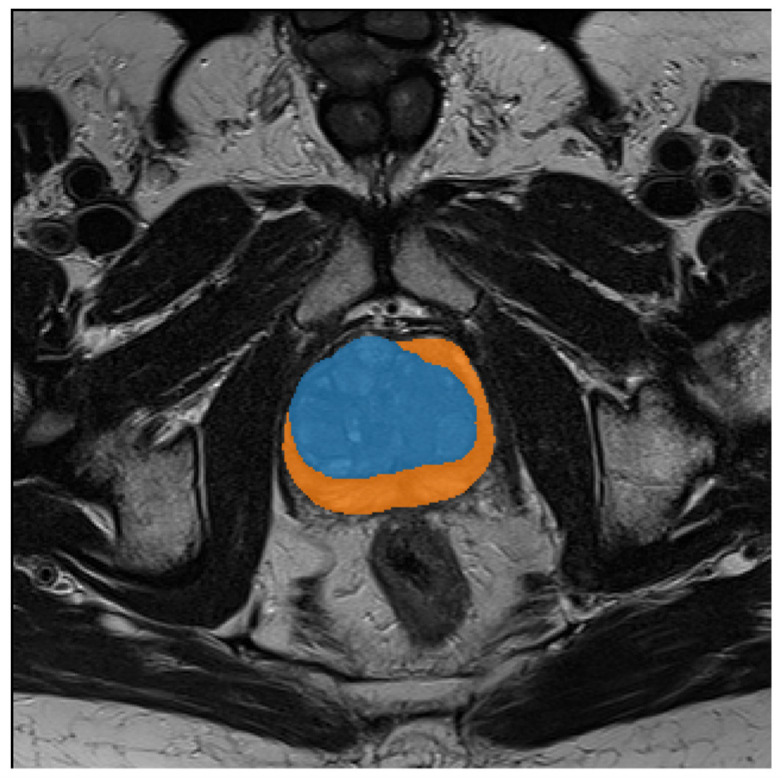
Visualization of the manual annotations prepared for each prostate MRI scan. The inner regions are depicted in blue, while orange shows the outer prostate region.

From the initial dataset, 326 MRI images of prostates collected from 41 patients were selected, in which the images were free of imaging artefacts and segmentation and visualization of the lesions were possible despite the rigorous PIRADS protocol. The number of projections per patient, which include the prostate, varies between 4 and 11 scans. Figure 1 shows the histogram of the number of MRI scans per patient. Each scan is supported with a manual annotation marking the inner (blue) and outer (orange) regions of the prostate, as depicted in Figure 2. For one patient, the outer prostate region was not annotated because this anatomical structure was absent. The mean age was 64.6 ± 9.8 with median age 65. The mean level of PSA in the cohort was 6.1 ± 3.9 with the median at 5.14. More detailed information on the cohort is given in Appendix A.

The image data with the corresponding segmentations are all publicly available on the website Zenodo [55].

### 2.3. Textural Features

The MaZda software was used to calculate the textural features for the annotated regions in the MRI scans. This approach was chosen due to the ease of setting the number of significant bits per image as a method parameter. This approach is not straightforward, necessitating the implementation of the *radiomics* Python library, which is also often used for textural feature determination. As we had access to several methods for texture description that may be preprocessed and parameterized in several ways, around 7000 features were calculated to describe a region. A brief overview of the applied methods is presented in this section. Since those methods are well known in the domain of image analysis and are also frequently used as MRI scan descriptors, we refer the reader to the MaZda manual for more details and formulas.

Before the textural features were calculated, the image color space was converted to YUV. Next, data normalization and quantization were undertaken if necessary. This step diminishes the brightness differences between the analyzed images, which could arise due to differences in the acquisition hardware. The system supports several normalization techniques, the details of which are given in Table 1, and also enables gray-level coding to a chosen number of bits. The preprocessed images were normalized in the region of interest (ROI) according to the methods shown in Table 2; the textural features were calculated using the following methods. The descriptors are derived only for the selected region: the inner prostate, outer prostate, or inner and outer prostate.

An image brightness histogram (Hist) is the simplest statistical description of the image content. It allows for the calculation of the following textural features: area, mean, variance, skewness, kurtosis, and percentiles. The gradient map features (Grad) describe the rapid illumination changes in small neighborhoods. This feature map is then transformed into a histogram, allowing us to obtain the following textural features: mean, variance, skewness, kurtosis, and non-zero elements. An autoregressive model (Arm) investigates the influence of neighboring pixels on each other. The method searches for the optimal solution by returning information about directional influence described by four theta parameters and an additional sigma parameter that conveys information about the error standard deviation.

The gray-level co-occurrence matrix method [32] is a more complex approach that concentrates on local neighborhoods. This matrix holds information about the counts of co-occurrence of pixel gray levels next to each other. Then, from this information, several textural features are computed: the angular second moment, contrast, correlation, sum of squares, invariance deformation moment, sum of averages, sum variance, entropy, difference of variance, and difference of entropy. The method acronym is GLCM followed by one letter indicating the direction of runs supplied by the value indicating the distance between pixels considered as neighbors: H—horizontal, V—vertical, Z—diagonal, as shown by the middle part of the letter, N—the other diagonal.

Unlike previously used approaches, the gray-level run-length matrix [56] describes the content by considering a larger region. Here, the image analysis approach reflects the way humans perceive images. The high-quality images have rapid color changes (short runs), while keeping the same colors next to each other (long runs) is characteristic of images of low quality. In this work, the GRLM acronym followed by one letter indicating the direction of runs (H, V, Z, or N) is given. The information about the run length for pixel gray values creates a base matrix from which several textural features are derived: area, short-run emphasis, long-run emphasis, gray-level non-uniformity, mean gray-level non-uniformity, run-length non-uniformity, mean run-length non-uniformity, and fraction.

In local binary patterns (LBPs) [57], the neighborhood of each pixel also influences its understanding. The pixel information is coded considering a circular neighborhood of 4, 8, or 12 pixels. A histogram is prepared from the generated codes, and each of its bins becomes a textural feature described by the LBP or circularity analysis method: over-complete, transition, center-symmetric. This is followed by the number of neighbors.

Another popular method for image description uses histograms of oriented gradients (HOGs) [58]. This technique divides the image into small blocks, for which the gradients are calculated. Their orientations are binned into histograms. For cells (regions composed of several blocks), the histograms are concatenated and normalized to become a feature vector. Using the MaZda software, the HOG method is calculated for the following numbers of bins: 4, 8, 16, and 32.

The frequency components in a local neighborhood are also analyzed using a Gabor transform (Gab). This method is parameterized with the Gaussian envelope, orientation, the period of the sinewave, and the magnitude. On the other hand, the discrete wavelet transform application is also used to derive image information. In this case, the Haar wavelet is used and its energies in the sub-bands become features.

Considering both the large number of methods that can be used to describe the content of MRI prostate scans and the ease of its parameterization, a set of around 7000 features was calculated for each image. Please see the feature naming convention given in Appendix B.

### 2.4. Multiple-Instance Learning with a Support Vector Machine

**Figure 3:**
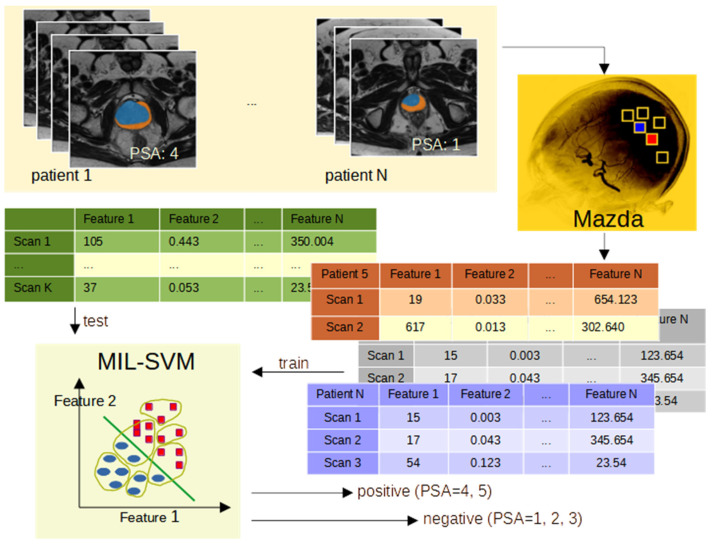
The overview of the presented approach.

In the present problem, several scans are described by one label. Therefore, multiple-instance learning seems to be the best approach to classification. This is a type of supervised learning; however, instead of having a separate label for each sample, all samples with one label are analyzed together and referred to as a bag. Then, the classifier decides that a bag of samples is negative when all samples are negative, and that it is positive when at least one sample within a bag is positive. When applying this method to our problem, the MIL approach returns a “healthy” label if all the MRI scans in the bag are negative, while it returns an “ill” label when at least one MRI scan depicts cancerous tissue. This approach reflects the fact that cancer might be noticed only in one scan, while the other parts are not infected. This approach works with the support vector machine as a binary classifier. For our purposes, the “mil” Python library was used. Figure 3 shows an overview of the presented approach.

## 3. Results

In this study, three sets of experiments were performed. Each of them was concerned with textural features calculated for different prostate regions annotated on the MRI scans, namely, the inner, outer, and inner and outer regions. Some of the textural features could not be calculated for all scans and have therefore been disregarded. For the rest of the scans, the procedure was as follows. First, the Pearson correlation between pairs of textural features was evaluated. Since the number of features is large (6678 features for the prostate inner region, 4898 for the prostate outer region, and 6718 for the total prostate region) in comparison to the number of samples (41, 40, and 40, respectively), it was decided to prepare the MIL-SVM models using only a pair of non-correlated features. Using a smaller number of representative features is also beneficial because it improves the SVM convergence. We assumed that pairs of features with a correlation higher than 50% should be disregarded (around 10% of all possible combinations). Then, the leave-one-out procedure was adopted to verify the model’s generality for each pair of textural features (giving around 60,000/54,000/64,000 possible pairs). This methodology assumes that the model is trained with n-1 samples, using one sample for testing, and that the experiment is repeated n times. The performance of the model was evaluated according to its accuracy, specificity, recall, and F1 score metrics. Both the leave-one-out procedure and SVM had a weight-balanced flag set, since there were 28 positive and 13 negative samples. For SVM, the linear kernel was applied with the regularization factor c = 10.

Table 3, Table 4 and Table 5 gather the 20 best results recorded for each type of experiment; the outcomes for the remaining pairs of features are attached as Supplementary Materials (Table S1). The highest scores are reached when the inner prostate region is considered (Table 3), with slightly worse outcomes for the whole prostate (Table 2); meanwhile, the outer region of the prostate seems to be slightly less significant (Table 4). However, these differences are very small. It is interesting to note that, depending on the region, different textural features play significant roles. This effect might be induced by the shape of the region of interest, as it is circular in the case of the inner prostate and whole prostate and elongated when only the outer part is analyzed. The elongation with a slight rotation of objects on MRI scans must have influenced the calculation of descriptors, as the computation of many of them depends on the chosen angle. Despite these differences and obstacles, very high F1 scores were recorded: 92.86%, 91.23%, and 89.80% for the inner, whole, and outer prostate regions, respectively.

As we can see from Table 3, the best differentiation between clinically significant and non-significant prostate cancers is achieved when the gray-level co-occurrence matrix summed variance feature is used along with Gabor wavelet magnitude. For the first textural feature, slightly better outcomes (92.86%) are attained when the image is preprocessed with min–max normalization (M) using only six significant bits to calculate the matrix in the horizontal direction (H) with stride equal to five. Changing the direction to diagonal (Z) with a slightly varying stride (4, 5), as well as using a different number of bits (4–8), diminishes the performance to 90.91%, while recall is maintained at the same level (92%) and the precision deteriorates from 92% to 89%. In all cases, the second textural feature is calculated for the original image using the Gabor wavelet with constant Gaussian envelope (24) in the horizontal direction and a sinewave period equal to 12. Since the dataset is highly imbalanced, the AUC was not calculated, as it works well only with balanced data and is too optimistic in other cases. Applying two textural features and concentrating on Gabor frequencies (YN7Gab12N6Mag and YD8Gab24H12Mag or YN8Gab12N6Mag and YD8Gab24H12Mag) also give very high F1 scores: 91% and 89%, respectively. The combination of the Gabor magnitude feature (YM8Gab12H6Mag) with histogram parameters (YS8HistDomn01) and other metrics calculated from the gray-level co-occurrence matrix (YM8GlcmZ5InvDfMom) also performs very well, with an F1 score around 88%.

When analyzing the whole prostate region, the best performance was recorded when combining Gabor frequency features with histogram percentiles, reaching an F1 score of 91.12% with recall of 89.66% and precision of 92.86%. Since there are similar outcomes, we can assume that using quantization (diminishing the number of input bits) in the case of Gabor wavelet calculation does not influence the performance, as similar results are achieved for data using from 4 up to 8 bits. Here, the horizontal orientation of a sinewave with period equal to 8 and Gaussian envelope equal to 16 was applied. The data were normalized using min–max transformation to calculate the histogram percentile using 8-bit data. The following results are derived mostly for pairs of features that focus on the image frequency description and not on some textural features, in an effort to express the human perception of the visual information.

The best outcomes for the outer prostate region are recorded when the correlation feature from the gray-level co-occurrence matrix is supported with the Gabor wavelet feature. Next, the combination of two features is used to describe the following texture frequencies. In the best case, the GLCM method is applied to min–max-normalized images; again, changing the number of significant bits (from 5 to 8) does not influence the F1 score—89.80%—with recall of 100% and precision of 81.48%. The second textural feature is derived from a normalized 8-bit image transformed with a Gabor filter with a Gaussian envelope of 24 radios, calculated in a diagonal orientation (Z) with a sinewave amplitude equal to 12.

## 4. Discussion

Also known as human kallikrein peptidase 3 (hK3), PSA is a member of the kallikrein gene family [59]. Ectopic PSA expression has been found at lower concentrations in malignant breast tissue, normal breast tissue, breast milk, and adrenal and kidney cancer. PSA is highly organ specific, as it is mainly produced by prostate epithelial cells. As evidenced by its imperfect performance as a diagnostic biomarker, PSA is not cancer specific and its values can be elevated in men with benign and malignant prostate diseases [60]. An increase in PSA is secondary to the loss of the barrier provided by the basal layer and the basal membranes of the prostate, which is a mandatory condition for the hormone to enter the circulation. However, loss of the barrier may occur in the case of different prostate diseases (BPH, prostatitis, prostate cancer) and in connection with prostate manipulation of the prostate (prostate massage, prostate biopsy) [61].

Screening for prostate cancer with the use of multiparametric MRI (mp-MRI) is still one of the most controversial topics in the urological literature [62]. Screening results reveal a significantly increased diagnosis of prostate cancer with the use of mp-MRI with detection of less advanced prostate cancer, however, with no overall survival benefit was observed [63]. National USA recommendations against PSA-based screening resulted in a reduction in the use of PSA for early detection and were associated with higher rates of advanced disease [64]. The inclusion of mp-MRI can improve a screening protocol, as it reduces the number of men who undergo biopsies while detecting more high- and intermediate-grade prostate cancer [65,66]. The IP1-PROSTAGRAM study (PSA > 3 ng/mL; MRI Prostate Imaging Reporting and Data System (PIRADS) > 2) showed the highest detection of prostate cancer by MRI compared to transrectal ultrasound-guided prostate biopsy (TRUS) in a population screening setting [65]. Moreover, standard TRUS is not reliable in detecting prostate cancer and the diagnostic yield of additional biopsies performed on hypoechoic lesions is negligible [67]. New sonographic modalities such as micro-Doppler, sonoelastography, or contrast-enhanced US provided promising preliminary findings, alone or combined into the so-called “multiparametric US” [68,69]. In the multiparametric US vs. multiparametric MRI to diagnose prostate cancer (CADMUS) trial, multiparametric US detected 4.3% fewer prostate cancer cases while submitting 11.1% more patients to biopsy than MRI [70]. The 68 m labeled prostate-specific antigen (PSA) positron emission tomography (PET PSMA) method is gaining increasing attention in the medical community as a method with increasing clinical potential [71,72]. It was proven that the diagnostic performance of PET PSMA is better than multiparametric magnetic resonance (mp-MRI) presented in clinical studies of intermediate-risk cancer, with diagnostic confidence reaching 80% in comparison to 60% for mp-MRI [73]. For advanced cancer, reported confidence was up to 99% in comparison to 87–97% reported for mp-MRI [74]. As PET PSMA gains increasing attention and influences treatment change in patients previously diagnosed with MR [75,76], there is a need to improve diagnostic techniques based on mp-MRI, especially in regard to the performance of 1.5 T scanners, as obtained results are less significant compared to those presented by 3T scanners [77,78].

Therefore, in asymptomatic patients with PSA values of 2–10 ng/mL, additional methods should be used to decide whether to refer the patient for a prostate biopsy, the result of which determines the treatment when cancer is diagnosed. These methods include cancer risk calculators as well as imaging-based diagnostics [79]. If PSA level is sufficiently high in correlation with the clinical picture (especially palpation methods), the margin for misleading diagnoses is very narrow [80]. However, in the many cases with intermediate levels of PSA, other diagnostic techniques, including diagnostic imaging, are needed to provide further support.

Magnetic resonance imaging (MRI) has been used for the non-invasive evaluation of the prostate and surrounding tissues since the 1980s. Advances in technology (both in software and hardware) have led to the development of multiparameter MRI (mp-MRI), which combines T2W anatomical imaging with functional and physiological assessments, including diffusion-based imaging (DWI) and its derivatives diffusion coefficient map (ADC) and dynamic MRI with contrast enhancement (DCE), and sometimes other techniques, such as in vivo MR proton spectroscopy [81].

MRI is currently a highly important step in the process of diagnosing suspected prostate cancer [23]. On the basis of over 30 years of experience gained in MR prostate studies, classification systems were created and are constantly updated [82]. However, it is widely acknowledged that a great deal of training is required to determine the image patterns behind prostate cancer, and there are still unclear images where diagnosis is not certain [83]. Therefore, clinical correlation is highly important, especially in relation to levels of PSA antigens [84].

Applying a pair of textural features to create an artificial intelligence model for the automatic detection of patients with high (≥4) scores of PSA as a cutoff value for determination of possible malignancy of the lesion is a promising approach, with an F1 score equal to 92%. In our experiments, we verified whether increasing the number of features could improve the outcome. In order to decrease the number of search possibilities, we concentrated on features that participated in generating models with F1 scores higher than 80%. For this approach, all those features were used as input, but principal component analysis was applied to select the most discriminative data. Nonetheless, these results did not outperform the presented ones, so are not discussed in detail.

## 5. Conclusions

The results presented are very promising, as the analysis of the MR images has shown a correlation with the biochemical levels of the cancer marker. This is an important step towards making the diagnostic imaging process more objective. The results obtained will be used in the future to develop the analysis in a larger group of patients. This could serve as a solid basis for the development of protocols for automated analysis of prostate magnetic resonance imaging to improve the assessment of prostate cancer in daily clinical practice and to reduce possible diagnostic errors.

### 5.1. Study Strengths

- Finding the correlation between MR image textural features (in accordance with PIRADS guidance) with proven levels of PSA and hence the risk of prostate cancer.
- Using a novel and unique approach in comparison to formerly applied methods of image analysis, with possible usefulness for automatic, computer-based systems of lesion detection.
- Evaluating magnetic resonance imaging of the prostate with the use of textural analysis; this constitutes another step towards the creation of fully automated protocols for a fast and effective diagnostic path for prostate cancer.

### 5.2. Study Weakness

- Some limitations of the present work concern the relatively small group of patients analyzed and incomplete data; resolving these issues would allow us to draw even more conclusions.

There is a correlation between the presence of increased PSA level and probability of cancer being identified with the aid of the results of diagnostic imaging analyses. Low PSA in patients with changes in the prostate image was found and the recognized findings in the MR images with no reflection in the increased PSA level might be misleading in practical settings.

## Figures and Tables

**Table 1:** Applied MR protocol in line with PIRADS guidance.

	TE	TR	FA	MX	VOX	FOV	CON	AV	PAT	DF	T
T2 (axial)	105	3320	160	256 × 320	0.6 × 0.6 × 2	200	2	4	2	0	300

TE—time echo, TR—relaxation time, FA—flip angle, MX—imaging matrix, VOX—voxel, FOV—field of view, CON—concentrations, AV—averages, PAT—Parallel acquisition, DF—distance factor, T—overall sequence time.

**Table 2:** Normalization methods accessible in MaZda software.

Normalization Method	Description
D	No normalization applied.
S	Gray levels normalized in the range <μ ± 3σ>, where μ—mean gray level value, σ—standard deviation.
M	Linear rescaling of the range to the minimal and maximal values.
N	Linear rescaling of the range defined by the 1st and 99th percentiles of the gray-level histogram.

**Table 3:** The best 20 pairs of features when the whole prostate region is used to calculate features.

Accuracy	Precision	Recall	F1 Score	Feature 1	Feature 2
0.878049	0.928571	0.896552	0.912281	YN6Gab16H8Mag	YM8HistPerc01
0.878049	0.928571	0.896552	0.912281	YN5Gab16H8Mag	YM8HistPerc01
0.878049	0.928571	0.896552	0.912281	YN8Gab16H8Mag	YM8HistPerc01
0.878049	0.928571	0.896552	0.912281	YN7Gab16H8Mag	YM8HistPerc01
0.878049	0.928571	0.896552	0.912281	YN4Gab16H8Mag	YM8HistPerc01
0.878049	0.892857	0.925926	0.909091	YN6Gab8N4Mag	YS8Gab24N12Mag
0.878049	0.892857	0.925926	0.909091	YS4ArmTeta2	YD8Gab24N12Mag
0.853659	0.857143	0.923077	0.888889	YLbpCs8n15	YM8HistPerc01
0.853659	0.892857	0.892857	0.892857	YM8HistPerc01	YS8Gab12Z6Mag
0.853659	0.892857	0.892857	0.892857	YN5ArmTeta4	YD8GradSkewness
0.853659	0.892857	0.892857	0.892857	YN7ArmTeta4	YD8GradSkewness
0.853659	0.892857	0.892857	0.892857	YN8ArmTeta4	YD8GradSkewness
0.853659	0.928571	0.866667	0.896552	YM4DwtHaarS2HL	YS8HistKurtosis
0.853659	0.857143	0.923077	0.888889	YN5Gab8N4Mag	YS8Gab24N12Mag
0.853659	0.892857	0.892857	0.892857	YS8GlcmZ4SumEntrp	YD8GradSkewness
0.853659	0.892857	0.892857	0.892857	YN8Gab8N4Mag	YS8Gab24N12Mag
0.853659	0.892857	0.892857	0.892857	YN7Gab8N4Mag	YS8Gab24N12Mag
0.853659	0.821429	0.958333	0.884615	YS8ArmTeta2	YD8Gab24N12Mag
0.853659	0.857143	0.923077	0.888889	YS6ArmTeta2	YD8Gab24N12Mag
0.853659	0.857143	0.923077	0.888889	YS5ArmTeta2	YD8Gab24N12Mag

**Table 4:** The best 20 pairs of features when the inner prostate region is used to calculate features.

Accuracy	Precision	Recall	F1 Score	Feature1	Feature2
0.902439	0.928571	0.928571	0.928571	YM6GlcmH5SumVarnc	YD8Gab24H12Mag
0.878049	0.892857	0.925926	0.909091	YN7Gab12N6Mag	YD8Gab24H12Mag
0.878049	0.892857	0.925926	0.909091	YM5GlcmZ4SumVarnc	YD8Gab24H12Mag
0.878049	0.892857	0.925926	0.909091	YM4GlcmZ4SumVarnc	YD8Gab24H12Mag
0.878049	0.892857	0.925926	0.909091	YM7GlcmZ4SumVarnc	YD8Gab24H12Mag
0.878049	0.892857	0.925926	0.909091	YM6GlcmZ4SumVarnc	YD8Gab24H12Mag
0.878049	0.892857	0.925926	0.909091	YM8GlcmZ4SumVarnc	YD8Gab24H12Mag
0.878049	0.892857	0.925926	0.909091	YM4GlcmH5SumVarnc	YD8Gab24H12Mag
0.878049	0.892857	0.925926	0.909091	YM5GlcmH5SumVarnc	YD8Gab24H12Mag
0.878049	0.892857	0.925926	0.909091	YM7GlcmH5SumVarnc	YD8Gab24H12Mag
0.853659	0.785714	1.000000	0.880000	YM8Gab12H6Mag	YS8HistDomn01
0.853659	0.857143	0.923077	0.888889	YN8Gab12N6Mag	YD8Gab24H12Mag
0.853659	0.857143	0.923077	0.888889	YM8GlcmZ5InvDfMom	YD8Gab24Z12Mag
0.853659	0.821429	0.958333	0.884615	YM4GlcmZ5SumVarnc	YD8Gab24H12Mag
0.853659	0.892857	0.892857	0.892857	YM4GlcmN2SumVarnc	YD8Gab24H12Mag
0.853659	0.857143	0.923077	0.888889	YM4GlcmH4SumVarnc	YD8Gab24H12Mag
0.853659	0.857143	0.923077	0.888889	YM5GlcmH4SumVarnc	YD8Gab24H12Mag
0.853659	0.857143	0.923077	0.888889	YM7GlcmH4SumVarnc	YD8Gab24H12Mag
0.853659	0.857143	0.923077	0.888889	YM6GlcmH4SumVarnc	YD8Gab24H12Mag
0.853659	0.857143	0.923077	0.888889	YM8GlcmH4SumVarnc	YD8Gab24H12Mag

**Table 5:** The best 20 pairs of features when the outer prostate region is used to calculate features.

Accuracy	Precision	Recall	F1 Score	Feature1	Feature2
0.875000	0.814815	1.000000	0.897959	YM7GlcmN2Correlat	YS8Gab24Z12Mag
0.875000	0.814815	1.000000	0.897959	YM8GlcmN2Correlat	YS8Gab24Z12Mag
0.875000	0.814815	1.000000	0.897959	YM6GlcmN2Correlat	YS8Gab24Z12Mag
0.875000	0.814815	1.000000	0.897959	YM5GlcmN2Correlat	YS8Gab24Z12Mag
0.850000	0.888889	0.888889	0.888889	YS7HogO8b2	YD8GradNonZeros
0.850000	0.888889	0.888889	0.888889	YN6Gab24N12Mag	YD8GradSkewness
0.850000	0.888889	0.888889	0.888889	YN7Gab24N12Mag	YD8GradSkewness
0.850000	0.888889	0.888889	0.888889	YN8Gab24N12Mag	YD8GradSkewness
0.850000	0.888889	0.888889	0.888889	YS4Gab24Z12Mag	YD8Gab16Z8Mag
0.850000	0.888889	0.888889	0.888889	YS6Gab24Z12Mag	YD8Gab16Z8Mag
0.850000	0.888889	0.888889	0.888889	YS7Gab24Z12Mag	YD8Gab16Z8Mag
0.850000	0.888889	0.888889	0.888889	YS8Gab24Z12Mag	YD8Gab16Z8Mag
0.850000	0.888889	0.888889	0.888889	YN7HistMaxm01	YS8Gab12Z6Mag
0.850000	0.777778	1.000000	0.875000	YM4GlcmN2Correlat	YS8Gab24Z12Mag
0.850000	0.814815	0.956522	0.880000	YM6GlcmH4InvDfMom	YD8Gab24N12Mag
0.850000	0.777778	1.000000	0.875000	YM7GlcmH4InvDfMom	YD8Gab24N12Mag
0.850000	0.888889	0.888889	0.888889	YN6GradNonZeros	YS8Gab16H8Mag
0.850000	0.814815	0.956522	0.880000	YN5GlcmV2Entropy	YD8HistKurtosis
0.825000	0.888889	0.857143	0.872727	YS8HogO8b2	YD8GradNonZeros
0.825000	0.814815	0.916667	0.862745	YN4Gab24V12Mag	YS8Gab12Z6Mag

## Data Availability

One hundred and fourteen T2-weighted MRI 3D images of the prostate with corresponding segmentations of transition and peripheral zones are available on the Zenodo website, https://doi.org/10.5281/zenodo.7676958.

## Supplementary Materials

The following supporting information can be downloaded at: https://www.mdpi.com/article/10.3390/jcm12082836/s1, Table S1. Prostaty results.

## Abbreviations

ADC	Apparent diffusion coefficient imaging
AI	Artificial intelligence
ANN	Artificial neural network
Arm	Autoregressive model
AUC	Area under curve
AV	Averages
BW	Bandwidth
CAD	Computer-aided diagnosis
CON	Concentrations
DCE	Dynamic contrast-enhanced MRI
DWI	Diffusion-based imaging
dw-MRI	Diffusion-weighted MRI
FA	Flip angle
FOV	Field of view
TF	Turbo factor
PAT	Parallel acquisition
DF	Distance factor
Gab	Gabor transform
GLCM	Grey-level co-occurrence matrix
Grad	Gradient map features
Hist	Brightness histogram
HOG	Histogram of oriented gradients
LBP	Local binary pattern
MIL	Multiple-instance learning
mp-MRI	Multiparameter MRI
MR	Magnetic resonance
MRI	Magnetic resonance imaging
MX	Imaging matrix
PIRADS	Prostate Imaging Reporting and Data System
PSA	Prostate-specific antigen
ROI	Region of interest
SVM	Support vector machine
T	Time
TE	Time echo
TF	Turbo factor
TR	Relaxation time
T2W-MRI	T2-weighted MRI
VOX	Voxel
YUV	Color model, Y—luminance, U—blue projection, V—red projection
TRUS	Transrectal ultrasound
US	Ultrasonography
CADMUS	Cancer Detection by Multiparametric Ultrasound of the Prostate
PET PSMA	PSA labeled positron emission tomography

## Appendix A. Dataset Detailed Description

Table A1 presents the basic information about the statistics of the patients’ age, PSA level, etc. in the dataset, while Table A1 gives detailed information for each patient.

**Table A1:** Basic dataset characteristics.

Patient count	41
Age (in years)	
Average	64.6 ± 9.8
Median	65
Range	39–85
PSA level	
Average	6.1 ± 3.9
Median	5.14
Range	0.39–19
Side	
Left	9 patients (22%)
Right	13 patients (32%)
Both	19 patients (46%)
Prostatitis	8 patients (20%)
BPH	23 patients (56%)

**Table A2:** Detailed description of the medical parameters of the examined cohort.

Patient No	Age	PSA Level	Side	Prostatitis	BPH
1	61	3.50	Left	Yes	Yes
2	62	1.75	Both	Yes	Yes
3	85	5.20	Right	No	Yes
4	63	7.50	Right	No	Yes
5	63	5.60	Right	No	Yes
6	69	6.70	Right	No	Yes
7	67	5.14	Right	No	No
8	78	19.00	Both	No	No
9	68	2.40	Left	Yes	Yes
10	44	3.15	Both	Yes	No
11	62	8.00	Both	No	No
12	62	8.10	Both	No	Yes
13	57	8.60	Both	No	Yes
14	61	8.80	Right	No	No
15	67	4.30	Both	No	No
16	51	9.90	Both	No	No
17	66	17.00	Left	No	No
18	70	5.90	Both	No	Yes
19	65	4.8	Right	No	No
20	70	4.9	Right	No	Yes
21	48	3.3	Both	No	No
22	64	3.4	Left	No	No
23	58	4.5	Both	Yes	No
24	66	2.7	Left	No	No
25	81	4.45	Left	No	Yes
26	62	17.00	Right	Yes	Yes
27	74	5.30	Both	No	Yes
28	58	0.39	Both	Yes	Yes
29	70	6.7	Right	No	Yes
30	77	8.1	Both	No	Yes
31	77	7	Both	No	Yes
32	64	5.2	Left	No	Yes
33	65	6.95	Both	No	Yes
34	55	3.9	Right	Yes	No
35	69	9.00	Both	No	Yes
36	62	3.60	Right	No	Yes
37	39	3.16	Both	No	No
38	50	3.85	Right	No	No
39	73	3.3	Left	No	Yes
40	81	4.5	Left	No	No
41	66	4	Both	No	No

## Appendix B. Feature-Naming Conventions

The textural feature names follow a convention whereby the description contains all of the information about the applied method and its parameters and preprocessing steps; the convention also ensures that the names are unique. The unique textural feature identities comprise the following:

- color component—Y always stands for the brightness component from the YUV color space;
- information on normalization, see Table 1;
- the number of bits used for quantization purposes;
- sliding window shape and size (if applied);
- the applied texture feature method;
- an acronym of the parameter;
- and the feature name abbreviation.

For instance, the feature name YM6GlcmH5SumVariance refers to an image converted to the YUV color space where the brightness component is analyzed. It is normalized using the minimum and maximum range of gray-scale pixel values (M) and coded on 6 bits. The gray-level co-occurrence matrix (GLCM) was applied to calculate the matrix in a horizontal (H) fashion, with the distance between adjoining pixels set to 5; finally, the sum of variance is the final feature in the acronym.

## References

[1] Culp M.B., Soerjomataram I., Efstathiou J.A., Bray F., Jemal A. (2020). Recent Global Patterns in Prostate Cancer Incidence and Mortality Rates. Eur. Urol..

[2] Wang L., Lu B., He M., Wang Y., Wang Z., Du L. (2022). Prostate Cancer Incidence and Mortality: Global Status and Temporal Trends in 89 Countries From 2000 to 2019. Front. Public Health.

[3] Mejak S.L., Bayliss J., Hanks S.D. (2013). Long Distance Bicycle Riding Causes Prostate-Specific Antigen to Increase in Men Aged 50 Years and Over. PLoS ONE.

[4] Ankerst D.P., Thompson I.M. (2006). Sensitivity and Specificity of Prostate-Specific Antigen for Prostate Cancer Detection with High Rates of Biopsy Verification. Arch. Ital. Urol. Androl. Organo Uff..

[5] Thompson I.M., Chi C., Ankerst D.P., Goodman P.J., Tangen C.M., Lippman S.M., Lucia M.S., Parnes H.L., Coltman C.A. (2006). Effect of Finasteride on the Sensitivity of PSA for Detecting Prostate Cancer. J. Natl. Cancer Inst..

[6] Damadian R. (1971). Tumor Detection by Nuclear Magnetic Resonance. Science.

[7] Steyn J.H., Smith F.W. (1982). Nuclear Magnetic Resonance Imaging of the Prostate. Br. J. Urol..

[8] Langlotz C.P. (1996). Benefits and Costs of MR Imaging of Prostate Cancer. Magn. Reson. Imaging Clin. N. Am..

[9] Potosky A.L. (1995). The Role of Increasing Detection in the Rising Incidence of Prostate Cancer. JAMA.

[10] Murphy G., Haider M., Ghai S., Sreeharsha B. (2013). The Expanding Role of MRI in Prostate Cancer. Am. J. Roentgenol..

[11] Haider M.A., van der Kwast T.H., Tanguay J., Evans A.J., Hashmi A.-T., Lockwood G., Trachtenberg J. (2007). Combined T2-Weighted and Diffusion-Weighted MRI for Localization of Prostate Cancer. Am. J. Roentgenol..

[12] Verma S., Turkbey B., Muradyan N., Rajesh A., Cornud F., Haider M.A., Choyke P.L., Harisinghani M. (2012). Overview of Dynamic Contrast-Enhanced MRI in Prostate Cancer Diagnosis and Management. Am. J. Roentgenol..

[13] Cauni V.M., Stanescu D., Tanase F., Mihai B., Persu C. (2021). Magnetic Resonance/Ultrasound Fusion Targeted Biopsy of the Prostate Can Be Improved by Adding Systematic Biopsy. Med. Ultrason..

[14] Frisbie J.W., Van Besien A.J., Lee A., Xu L., Wang S., Choksi A., Afzal M.A., Naslund M.J., Lane B., Wong J. (2022). PSA Density Is Complementary to Prostate MP-MRI PI-RADS Scoring System for Risk Stratification of Clinically Significant Prostate Cancer. Prostate Cancer Prostatic Dis..

[15] Wong T., Schieda N., Sathiadoss P., Haroon M., Abreu-Gomez J., Ukwatta E. (2021). Fully Automated Detection of Prostate Transition Zone Tumors on T2-Weighted and Apparent Diffusion Coefficient (ADC) Map MR Images Using U-Net Ensemble. Med. Phys..

[16] Gaur S., Lay N., Harmon S.A., Doddakashi S., Mehralivand S., Argun B., Barrett T., Bednarova S., Girometti R., Karaarslan E. (2018). Can Computer-Aided Diagnosis Assist in the Identification of Prostate Cancer on Prostate MRI? A Multi-Center, Multi-Reader Investigation. Oncotarget.

[17] Ishioka J., Matsuoka Y., Uehara S., Yasuda Y., Kijima T., Yoshida S., Yokoyama M., Saito K., Kihara K., Numao N. (2018). Computer-Aided Diagnosis of Prostate Cancer on Magnetic Resonance Imaging Using a Convolutional Neural Network Algorithm. BJU Int..

[18] Hambrock T., Vos P.C., Hulsbergen-van de Kaa C.A., Barentsz J.O., Huisman H.J. (2013). Prostate Cancer: Computer-Aided Di-agnosis with Multiparametric 3-T MR Imaging—Effect on Observer Performance. Radiology.

[19] Reda I., Khalil A., Elmogy M., Abou El-Fetouh A., Shalaby A., Abou El-Ghar M., Elmaghraby A., Ghazal M., El-Baz A. (2018). Deep Learning Role in Early Diagnosis of Prostate Cancer. Technol. Cancer Res. Treat..

[20] Haralick R.M. (1979). Statistical and Structural Approaches to Texture. Proc. IEEE.

[21] Kociołek M., Strzelecki M., Klepaczko A., Pietka E., Badura P., Kawa J., Wieclawek W. (2019). Functional Kidney Analysis Based on Textured DCE-MRI Images. Information Technology in Biomedicine.

[22] Szczypiński P.M., Strzelecki M., Materka A., Klepaczko A., Kącki E., Rudnicki M., Stempczyńska J. (2009). MaZda—The Software Package for Textural Analysis of Bio-medical Images. Computers in Medical Activity.

[23] de Rooij M., Israël B., Tummers M., Ahmed H.U., Barrett T., Giganti F., Hamm B., Løgager V., Padhani A., Panebianco V. (2020). SUR/ESUI consensus statements on multi-parametric MRI for the detection of clinically significant prostate cancer: Quality requirements for image acquisition, interpretation and radiologists’ training. Eur. Radiol..

[24] Boesen L. (2017). Multiparametric MRI in Detection and Staging of Prostate Cancer. Dan. Med. Bull..

[25] Van Booven D.J., Kuchakulla M., Pai R., Frech F.S., Ramasahayam R., Reddy P., Parmar M., Ramasamy R., Arora H. (2021). A Systematic Review of Artificial Intelligence in Prostate Cancer. Res. Rep. Urol..

[26] Harmon S.A., Tuncer S., Sanford T., Choyke P.L., Türkbey B. (2019). Artificial Intelligence at the Intersection of Pathology and Radiology in Prostate Cancer. Diagn. Interv. Radiol. Ank. Turk..

[27] Telecan T., Andras I., Crisan N., Giurgiu L., Căta E.D., Caraiani C., Lebovici A., Boca B., Balint Z., Diosan L. (2022). More than Meets the Eye: Using Textural Analysis and Artificial Intelligence as Decision Support Tools in Prostate Cancer Diag-nosis—A Systematic Review. J. Pers. Med..

[28] Patel N., Henry A., Scarsbrook A. (2019). The Value of MR Textural Analysis in Prostate Cancer. Clin. Radiol..

[29] Liu X., Faes L., Kale A.U., Wagner S.K., Fu D.J., Bruynseels A., Mahendiran T., Moraes G., Shamdas M., Kern C. (2019). A Comparison of Deep Learning Performance against Health-Care Professionals in Detecting Diseases from Medical Imaging: A Systematic Review and Meta-Analysis. Lancet Digit. Health.

[30] Wildeboer R.R., van Sloun R.J.G., Wijkstra H., Mischi M. (2020). Artificial Intelligence in Multiparametric Prostate Cancer Imaging with Focus on Deep-Learning Methods. Comput. Methods Programs Biomed..

[31] Wibmer A., Hricak H., Gondo T., Matsumoto K., Veeraraghavan H., Fehr D., Zheng J., Goldman D., Moskowitz C., Fine S.W. (2015). Haralick Texture Analysis of Prostate MRI: Utility for Differentiating Non-Cancerous Prostate from Prostate Cancer and Differentiating Prostate Cancers with Different Gleason Scores. Eur. Radiol..

[32] Haralick R.M., Shanmugam K., Dinstein I. (1973). Textural Features for Image Classification. IEEE Trans. Syst. Man Cybern..

[33] Nketiah G., Elschot M., Kim E., Teruel J.R., Scheenen T.W., Bathen T.F., Selnæs K.M. (2017). T2-Weighted MRI-Derived Textural Features Reflect Prostate Cancer Aggressiveness: Preliminary Results. Eur. Radiol..

[34] Gnep K., Fargeas A., Gutiérrez-Carvajal R.E., Commandeur F., Mathieu R., Ospina J.D., Rolland Y., Rohou T., Vincendeau S., Hatt M. (2017). Haralick Textural Features on T2 -Weighted MRI Are Associated with Biochemical Recurrence Following Radiotherapy for Peripheral Zone Prostate Cancer. J. Magn. Reson. Imaging.

[35] Baek T.W., Kim S.H., Park S.J., Park E.J. (2020). Texture Analysis on Bi-Parametric MRI for Evaluation of Aggressiveness in Patients with Prostate Cancer. Abdom. Radiol. N. Y..

[36] Xiong H., He X., Guo D. (2021). Value of MRI Texture Analysis for Predicting High-Grade Prostate Cancer. Clin. Imaging.

[37] He X., Xiong H., Zhang H., Liu X., Zhou J., Guo D. (2021). Value of MRI Texture Analysis for Predicting New Gleason Grade Group. Br. J. Radiol..

[38] Damascelli A., Gallivanone F., Cristel G., Cava C., Interlenghi M., Esposito A., Brembilla G., Briganti A., Montorsi F., Castiglioni I. (2021). Advanced Imaging Analysis in Prostate MRI: Building a Radiomic Signature to Predict Tumor Aggres-siveness. Diagnostics.

[39] Giannini V., Mazzetti S., Defeudis A., Stranieri G., Calandri M., Bollito E., Bosco M., Porpiglia F., Manfredi M., De Pascale A. (2021). A Fully Automatic Artificial Intelligence System Able to Detect and Characterize Prostate Cancer Using Mul-tiparametric MRI: Multicenter and Multi-Scanner Validation. Front. Oncol..

[40] Li M., Yang L., Yue Y., Xu J., Huang C., Song B. (2020). Use of Radiomics to Improve Diagnostic Performance of PI-RADS v2.1 in Prostate Cancer. Front. Oncol..

[41] Bonekamp D., Kohl S., Wiesenfarth M., Schelb P., Radtke J.P., Götz M., Kickingereder P., Yaqubi K., Hitthaler B., Gählert N. (2018). Radiomic Machine Learning for Characterization of Prostate Lesions with MRI: Comparison to ADC Values. Radiology.

[42] Wang J., Wu C.-J., Bao M.-L., Zhang J., Wang X.-N., Zhang Y.-D. (2017). Machine Learning-Based Analysis of MR Radiomics Can Help to Improve the Diagnostic Performance of PI-RADS v2 in Clinically Relevant Prostate Cancer. Eur. Radiol..

[43] Min X., Li M., Dong D., Feng Z., Zhang P., Ke Z., You H., Han F., Ma H., Tian J. (2019). Multi-Parametric MRI-Based Radiomics Signature for Discriminating between Clinically Significant and Insignificant Prostate Cancer: Cross-Validation of a Machine Learning Method. Eur. J. Radiol..

[44] Zhang Y., Chen W., Yue X., Shen J., Gao C., Pang P., Cui F., Xu M. (2020). Development of a Novel, Multi-Parametric, MRI-Based Radiomic Nomogram for Differentiating between Clinically Significant and Insignificant Prostate Cancer. Front. Oncol..

[45] Song Y., Zhang Y.-D., Yan X., Liu H., Zhou M., Hu B., Yang G. (2018). Computer-Aided Diagnosis of Prostate Cancer Using a Deep Convolutional Neural Network from Multiparametric MRI. J. Magn. Reson. Imaging.

[46] de Vente C., Vos P., Hosseinzadeh M., Pluim J., Veta M. (2021). Deep Learning Regression for Prostate Cancer Detection and Grading in Bi-Parametric MRI. IEEE Trans. Biomed. Eng..

[47] Liu Y., Zheng H., Liang Z., Miao Q., Brisbane W.G., Marks L.S., Raman S.S., Reiter R.E., Yang G., Sung K. (2021). Textured-Based Deep Learning in Prostate Cancer Classification with 3T Multiparametric MRI: Comparison with PI-RADS-Based Classifica-tion. Diagnostics.

[48] Schelb P., Kohl S., Radtke J.P., Wiesenfarth M., Kickingereder P., Bickelhaupt S., Kuder T.A., Stenzinger A., Hohenfellner M., Schlemmer H.-P. (2019). Classification of Cancer at Prostate MRI: Deep Learning versus Clinical PI-RADS Assessment. Radiology.

[49] Kwak J.T., Xu S., Wood B.J., Turkbey B., Choyke P.L., Pinto P.A., Wang S., Summers R.M. (2015). Automated Prostate Cancer Detection Using T2-Weighted and High-b-Value Diffusion-Weighted Magnetic Resonance Imaging. Med. Phys..

[50] Zhao K., Wang C., Hu J., Yang X., Wang H., Li F., Zhang X., Zhang J., Wang X. (2015). Prostate Cancer Identification: Quantitative Analysis of T2-Weighted MR Images Based on a Back Propagation Artificial Neural Network Model. Sci. China Life Sci..

[51] Doyle S., Hwang M., Shah K., Madabhushi A., Feldman M., Tomaszeweski J. Automated Grading of Prostate Cancer Using Architectural and Textural Image Features. Proceedings of the 2007 4th IEEE International Symposium on Biomedical Imaging: From Nano to Macro.

[52] Sidhu H.S., Benigno S., Ganeshan B., Dikaios N., Johnston E.W., Allen C., Kirkham A., Groves A.M., Ahmed H.U., Em-berton M. (2017). Textural Analysis of Multiparametric MRI Detects Transition Zone Prostate Cancer. Eur. Radiol..

[53] Niu X.-K., Chen Z.-F., Chen L., Li J., Peng T., Li X. (2018). Clinical Application of Biparametric MRI Texture Analysis for Detection and Evaluation of High-Grade Prostate Cancer in Zone-Specific Regions. Am. J. Roentgenol..

[54] World Medical Association (2019). Issue Information-Declaration of Helsinki. J. Bone Miner. Res..

[55] Gibala S., Obuchowicz R., Lasek J., Schneider Z., Piorkowski A., Pociask E., Nurzynska K. (2023). Prostate MRI T2-Weighted Images with Peripherial and Trasition Zone Segmentations Including Corresponding PIRADS and PSA Values. https://zenodo.org/record/7676958#.ZDesiPZByUl.

[56] Galloway M.M. (1975). Texture Analysis Using Gray Level Run Lengths. Comput. Graph. Image Process..

[57] Ojala T., Pietikainen M., Maenpaa T. (2002). Multiresolution Gray-Scale and Rotation Invariant Texture Classification with Local Binary Patterns. IEEE Trans. Pattern Anal. Mach. Intell..

[58] Dalal N., Triggs B. Histograms of Oriented Gradients for Human Detection. Proceedings of the 2005 IEEE Computer Society Conference on Computer Vision and Pattern Recognition (CVPR’05).

[59] McCormack R.T., Wang T.J., Rittenhouse H.G., Wolfert R.L., Finlay J.A., Lilja H., Okoloff R.L., Oesterling J.E. (1995). Molecular Forms of Prostate-Specific Antigen and the Human Kallikrein Gene Family: A New Era. Urology.

[60] Partin A.W., Carter H.B., Chan D.W., Epstein J.I., Oesterling J.E., Rock R.C., Weber J.P., Walsh P.C. (1990). Prostate Specific Antigen in the Staging of Localized Prostate Cancer: Influence of Tumor Differentiation, Tumor Volume and Benign Hy-perplasia. J. Urol..

[61] Morote Robles J., Ruibal Morell A., Palou Redorta J., de Torres Mateos J.A., Soler Roselló A. (1988). Clinical Behavior of Prostatic Specific Antigen and Prostatic Acid Phosphatase: A Comparative Study. Eur. Urol..

[62] Etzioni R., Gulati R., Cooperberg M.R., Penson D.M., Weiss N.S., Thompson I.M. (2013). Limitations of basing screening policies on screening trials: The US Preventive Services Task Force and Prostate Cancer Screening. Med. Care.

[63] Ilic D., Djulbegovic M., Jung J.H., Hwang E.C., Zhou Q., Cleves A., Agoritsas T., Dahm P. (2018). Prostate cancer screening with prostate-specific antigen (PSA) test: A systematic review and meta-analysis. BMJ.

[64] Eklund M., Jäderling F., Discacciati A., Bergman M., Annerstedt M., Aly M., Glaessgen A., Carlsson S., Grönberg H., Nordström T. (2021). MRI-Targeted or Standard Biopsy in Prostate Cancer Screening. N. Engl. J. Med..

[65] Eldred-Evans D., Ahmed H.U. (2021). Population-Based Prostate Cancer Screening with Magnetic Resonance Imaging or Ultrasonography: The IP1-PROSTAGRAM Study. JAMA Oncol..

[66] Van Poppel H., Hogenhout R., Albers P., Bergh R.C.V.D., Barentsz J.O., Roobol M.J. (2021). Early Detection of Prostate Cancer in 2020 and Beyond: Facts and Recommendations for the European Union and the European Commission. Eur. Urol..

[67] Rouviere O., Puech P., Renard-Penna R., Claudon M., Roy C., Mège-Lechevallier F., Decaussin-Petrucci M., Dubreuil-Chambardel M., Magaud L., Remontet L. (2019). Use of prostate systematic and targeted biopsy on the basis of multiparametric MRI in biopsy-naive patients (MRI-FIRST): A prospective, multicentre, paired diagnostic study. Lancet Oncol..

[68] Correas J.M., Halpern E.J., Barr R.G., Ghai S., Walz J., Bodard S., Dariane C., de la Rosette J. (2021). Advanced ultrasound in the diagnosis of prostate cancer. World J. Urol..

[69] Mannaerts C.K., Engelbrecht M.R., Postema A.W., van Kollenburg R.A., Hoeks C.M., Savci-Heijink C.D., Van Sloun R.J.G., Wildeboer R.R., De Reijke T.M., Mischi M. (2020). Detection of clinically significant prostate cancer in biopsy-naïve men: Direct comparison of systematic biopsy, multiparametric MRI- and contrast-ultrasound-dispersion imaging-targeted biopsy. BJU Int..

[70] Grey A.D.R., Scott R., Shah B., Acher P., Liyanage S., Pavlou M., Omar R., Chinegwundoh F., Patki P., Shah T.T. (2022). Multiparametric ultrasound versus multiparametric MRI to diagnose prostate cancer (CADMUS): A prospective, multicentre, paired-cohort, confirmatory study. Lancet Oncol..

[71] Zschaeck S., Andela S.B., Amthauer H., Furth C., Rogasch J.M., Beck M., Hofheinz F., Huang K. (2022). Correlation Between Quantitative PSMA PET Parameters and Clinical Risk Factors in Non-Metastatic Primary Prostate Cancer Patients. Front. Oncol..

[72] Lisney A.R., Leitsmann C., Strauß A., Meller B., Bucerius J.A., Sahlmann C.O. (2022). The Role of PSMA PET/CT in the Primary Diagnosis and Follow-Up of Prostate Cancer-A Practical Clinical Review. Cancers.

[73] Zhou C., Tang Y., Deng Z., Yang J., Zhou M., Wang L., Hu S. (2022). Comparison of 68Ga-PSMA PET/CT and multiparametric MRI for the detection of low- and intermediate-risk prostate cancer. EJNMMI Res..

[74] Regmi S.K., Sathianathen N., Stout T.E., Konety B.R. (2021). MRI/PET Imaging in elevated PSA and localized prostate cancer: A narrative review. Transl. Androl. Urol..

[75] Perera M., Papa N., Christidis D., Wetherell W., Hofman M.S., Murphy D.G., Bolton D., Lawrentschuk N. (2016). Sensitivity, Specificity, and Predictors of Positive 68Ga-Prostate-specific Membrane Antigen Positron Emission Tomography in Advanced Prostate Cancer: A Systematic Review and Meta-analysis. Eur. Urol..

[76] van Kalmthout L.W.M., van Melick H.H.E., Lavalaye J., Meijer R.P., Kooistra A., de Klerk J.M.H., Braat A.J.A.T., Kaldeway H.P., de Bruin P.C., de Keizer B. (2020). Prospective Validation of Gallium-68 Prostate Specific Membrane Antigen-Positron Emission Tomography/Computerized Tomography for Primary Staging of Prostate Cancer. J. Urol..

[77] Beyersdorff D., Taymoorian K., Knösel T., Schnorr D., Felix R., Hamm B., Bruhn H. (2005). MRI of prostate cancer at 1.5 and 3.0 T: Comparison of image quality in tumor detection and staging. Am. J. Roentgenol..

[78] Virarkar M., Szklaruk J., Diab R., Bassett R., Bhosale P. (2022). Diagnostic value of 3.0 T versus 1.5 T MRI in staging prostate cancer: Systematic review and meta-analysis. Pol. J. Radiol..

[79] (2022). EAU Pocket Guidelines. Proceedings of the EAU Annual Congress, Milan, Italy, 10–13 March 2023.

[80] Omri N., Kamil M., Alexander K., Alexander K., Edmond S., Ariel Z., David K., Gilad A.E., Azik H. (2020). Association between PSA Density and Pathologically Significant Prostate Cancer: The Impact of Prostate Volume. Prostate.

[81] American College of Radiology ACR Appropriateness Criteria^®^. https://acsearch.acr.org/list.

[82] Beyer T., Schlemmer H.-P., Weber M.-A., Thierfelder K.M. (2021). PI-RADS 2.1—Image Interpretation: The Most Important Updates and Their Clinical Implications. ROFO. Fortschr. Geb. Rontgenstr. Nuklearmed..

[83] Turkbey B., Oto A. (2021). Factors Impacting Performance and Reproducibility of PI-RADS. Can. Assoc. Radiol. J..

[84] Gündoğdu E., Emekli E., Kebapçı M. (2020). Evaluation of Relationships between the Final Gleason Score, PI-RADS v2 Score, ADC Value, PSA Level, and Tumor Diameter in Patients That Underwent Radical Prostatectomy Due to Prostate Cancer. Radiol. Med..

